# A Case of Glioblastoma, Isocitrate Dehydrogenase Wild Type, With Widely Disseminated Osseous Metastasis

**DOI:** 10.7759/cureus.28803

**Published:** 2022-09-05

**Authors:** Brianna Conte, Benjamin J Rich, Sakir H Gultekin, Gregory Azzam, Maria Del Pilar Guillermo Prieto Eibl

**Affiliations:** 1 Radiation Oncology, University of Miami Miller School of Medicine, Miami, USA; 2 Pathology, University of Miami Miller School of Medicine, Miami, USA; 3 Radiaiton Oncology, University of Miami Miller School of Medicine, Miami, USA; 4 Neuro-Oncology, University of Miami Miller School of Medicine, Miami, USA

**Keywords:** concurrent chemo-radiation, bone metastases, extracranial metastases, glioblastoma, glioblastoma multiforme, metastatic glioblastoma, glioblastoma idh-wildtype

## Abstract

Glioblastoma, isocitrate dehydrogenase (IDH) wild type, is an aggressive primary brain malignancy with a poor prognosis, despite treatment including surgery, chemotherapy, and radiation therapy. Few patients with glioblastoma develop metastasis outside the neuroaxis, likely due to disease progression in the brain prior to extraneural dissemination. The driving mutations of tumors in patients with extraneural metastases are not well described. In this case, we present a severe case of extraneural metastatic glioblastoma, as well as the genetic mutations of the tumor.

## Introduction

Glioblastoma, isocitrate dehydrogenase (IDH) wild type, is an aggressive primary brain malignancy with median survival from diagnosis of 14-21 months with optimal treatment including surgery, chemotherapy, and radiation therapy, although the prognosis is much less for patients with metastases [1]. While glioblastoma is usually fatal due to local intracranial progression, about 0.5-2% of patients with glioblastoma will develop metastasis outside the neuroaxis [2]. Glioblastomas can metastasize extracranially, even in the absence of prior surgical manipulation, possibly due to hematogenous spread [3-5]. Due to their rarity, glioblastomas with extraneural metastasis have been limited to case reports or small case series with documented metastasis to bones, liver, lymph nodes, kidneys, adrenal glands, and lungs [6]. However, the documented cases of glioblastoma with extraneural metastasis report only limited metastatic disease. In this report, we report the case of a 55-year-old man with recurrent glioblastoma, IDH wild type, with the widely disseminated osseous disease.

## Case presentation

A 55-year-old male was diagnosed with glioblastoma, IDH wild type, of the right temporal and occipital lobes. Magnetic resonance imaging (MRI) of the brain at diagnosis demonstrated a 6.3 cm heterogeneously enhancing mass in the right temporal lobe extending toward the medial occipital lobe. The patient underwent a right craniotomy with subtotal tumor resection. Pathology of the tumor revealed a glioblastoma, IDH wild type. The O6-methyguanine-DNA methyltransferase (MGMT) promoter was unmethylated. Genomic tumor testing (Foundation Medicine, Cambridge MA) demonstrated an FGFR3-TACC3 fusion, mutations in the TERT gene promoter (124C>T) and the PIK3R1 gene (W583del) as well as loss of CDKN2A/B, FAS, MTAP, and PTEN. The patient underwent standard-of-care postoperative chemoradiation consisting of 60 Gy in 30 fractions with concurrent temozolomide at an outside institution. Post-chemoradiation imaging demonstrated disease progression despite treatment and the patient was started on bevacizumab. A follow-up MRI of the brain with perfusion completed a month later showed a persistent enhancing mass in the right occipital and temporal lobe consistent with tumor progression.

**Figure 1:**
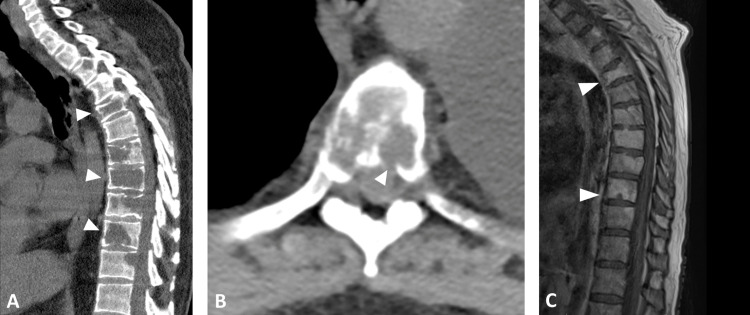
A. CT of thoracic spine, sagittal view, with white arrows depicting metastases. B. CT of thoracic spine, axial view, with white arrows depicting metastases. C. T1-weighted, post-contrast MRI image of the thoracic spine with white arrows depicting metastases. CT, computed tomography; MRI, magnetic resonance imaging.

The patient was admitted with upper back pain five weeks later, seven months after diagnosis. Imaging revealed multiple lytic-appearing cervical, thoracic, and lumbar vertebral body lesions (Figure 1). There were also lytic-appearing lesions in the left scapula and right eighth and 10th ribs. These lesions were not apparent on imaging at diagnosis. The patient underwent a biopsy of a lesion at L3, which revealed metastatic glioblastoma, morphologically identical to the patient's previously resected intracranial specimen with a proliferation index of approximately 30% by Ki-67 stain. The patient was subsequently placed on erdafitinib.

**Figure 2:**
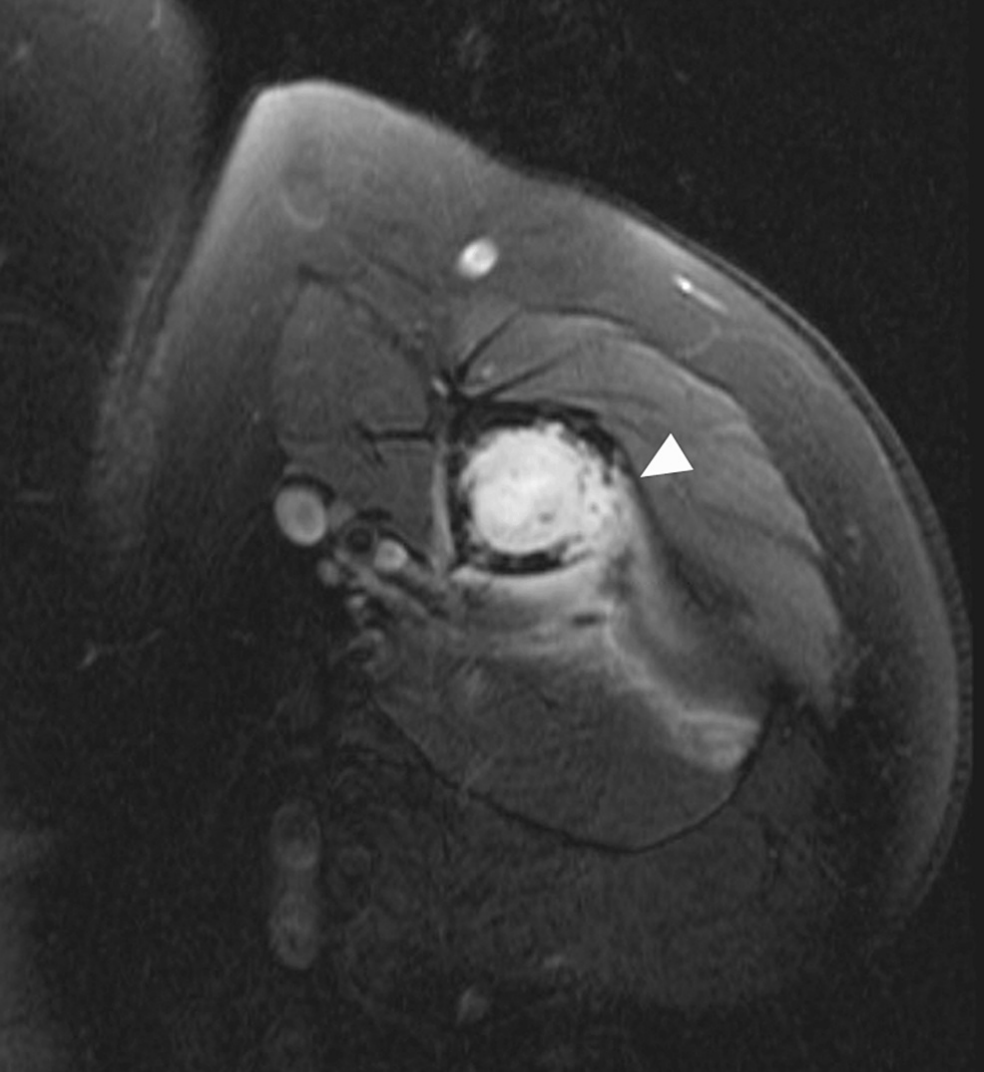
T2-weighted, fat-suppressed MRI image of the left humerus with white arrow demonstrating osseous metastases. MRI, magnetic resonance imaging.

**Figure 3:**
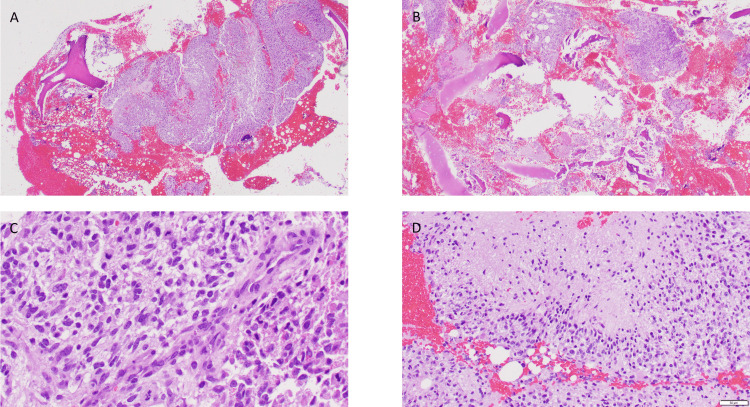
A. Pathology of humeral head. B. Low microscopic power of the lesion with fragments of bone and cartilage and adjacent fresh blood. C. Glioblastoma with mitotic figures, fibrillary background, and vascular proliferation. D. Glioblastoma with palisading necrosis.

The patient then transferred care to our institution. At the time of admission, he presented with a hemoglobin of 15.0 g/dL, hematocrit of 44.1%, and a platelet count of 197 k/uL. His white blood cell count was 10.4 k/uL with 5.2% neutrophils, 7.0% lymphocytes, 5.5% monocytes, 0.8% eosinophils, and 0.2% basophils. Further work-up demonstrated bilateral humeral lytic lesions measuring 5.8 cm on the right and 7 cm on the left (Figure 2). The patient completed numerous CTs, MRIs, and a full-body nuclear medicine bone scan which showed no evidence of a secondary tumor, confirming the diagnosis of metastatic glioblastoma based on Krishan’s modification of Weiss criteria [7]. The patient was treated with bilateral intramedullary nailing. Pathology again demonstrated glioblastoma with bone involvement (Figure 3). Erdafitinib was stopped due to worsening transaminitis, and the patient resumed bevacizumab. The patient was treated with palliative radiation therapy to the thoracic spine (20 Gy in 5 fractions) due to concerns about an impending cord compression. He also received a palliative course of re-irradiation (25 Gy in 5 fractions) to the progressing brain mass for symptomatic control. A pleural effusion subsequently developed. Analysis of pleural fluid with cytology found atypical cells, but a definitive diagnosis could not be rendered. The patient’s condition deteriorated and he died one month later, 10 months after diagnosis, of extracranial disease progression with pulmonary effusions the probable cause of death.

## Discussion

Glioblastoma metastases outside the neural axis are rare, and their description is limited to case reports and small case series. Glioblastoma metastasizes through the hematogenous spread and has been reported in the bone, lungs, lymph nodes, and visceral organs [3,8]. Although it has been hypothesized that surgical manipulation provides glioblastomas with hematogenous access, several reports of metastatic disease prior to surgery refute this [4,5]. While still exceptionally rare, the frequency of extraneural glioblastoma metastasis is becoming more common with the lengthening survival of glioblastoma patients [9,10]. The progression to extraneural metastasis is currently estimated to develop in 2% of patients with glioblastoma [2], but usually involves just a few extraneural sites [3]. This report of a patient with widespread, disseminated osseous metastasis from glioblastoma represents, to our knowledge, the most extensive metastatic glioblastoma reported in the literature. 

An etiology for the occurrence of glioblastoma metastasis is not known. One hypothesis is that a disruption of the blood-brain barrier by surgical manipulation leads to cell shedding outside of the brain. For example, cases have reported metastatic glioblastoma following placement of a ventriculoperitoneal shunt [11] or stereotactic biopsy [12]. However, one circulating tumor cell analysis of glioblastoma patients revealed that 20% of patients with glioblastoma had circulating tumor cells and showed no statistically significant correlation between a prior craniotomy and the presence of tumor cells circulating in the bloodstream [13]. Furthermore, an autopsy series of patients with glioblastoma found that 25% had evidence of spinal metastasis [14]. This may explain the case studies citing metastases without prior surgery [3-5]. In mice models, glioblastoma tumors grow outside of the central nervous system as well as transplanted carcinomas [15]. Therefore, the rarity of glioblastoma metastasis may be due to the limited overall survival of patients with glioblastoma due to intracranial progression, prior to the development of symptomatic extraneural metastasis.

Cases of metastatic glioblastoma have been seen in systemic vasculature, bones, the liver, the lungs, lymph nodes, and the neck [3]. In a review of 72 cases with extraneural metastases of astrocytomas and glioblastomas, metastases were present in each of the following sites: 43 (59.7%) lungs and pleura, 37 (51.4%) lymph nodes, 22 (30.5%) bone, 16 (22.2%) liver [16]. Less than 10% of cases had metastasis in the heart, adrenal gland, kidneys, mediastinum, pancreas, thyroid gland, and peritoneum [16]. Metastatic spread occurs through hematological or lymphogeneous tracks classically but has also been seen along cerebrospinal pathways [17].

The present case is one of the most severe cases of extraneural metastatic glioblastoma reported. Metastatic disease infiltrated almost all vertebrae of the spinal column as well as the bilateral humeri. Extensive metastases are suggestive of a highly aggressive tumor, early escape from the cranial space, or a combination of factors [18]. Our patient’s overall survival from diagnosis was 10 months but under three months from diagnosis of osseous metastasis. There is variable data on the association of extraneural metastasis in glioblastoma patients, with an overall survival range of 10-19 months from diagnosis of initial tumor [3,8]. Although the prognosis of patients with extraneural metastasis is poor, some reports suggest that survival can be prolonged to 10 months, with the intracranial disease more important for dictating survival [19].

Despite the poor prognosis associated with extraneural disease, our patient was treated aggressively after his diagnosis of extraneural disease. For example, he received bilateral intramedullary nailing for his humeral metastasis. Systemic therapy has been shown to control extraneural disease [20,21], but given the patient’s widespread disease and intracranial progression, he was unlikely to enjoy durable control. Hospice care is an appropriate treatment option in most patients with extraneural glioblastoma.

The genes driving metastasis of glioblastoma tumors are not known. A higher prevalence of mutations has been reported in tumor suppressors TP53 and RB in patients with metastatic extraneural glioblastoma [6,8]. Our patient’s tumor had an FGFR3-TACC3 fusion, mutations in the TERT gene promoter (124C>T) and the PIK3R1 gene (W583del), as well as loss of CDKN2A/B, FAS, MTAP, and PTEN.

Therapeutic advances have increased long-term survival for patients with glioblastoma [1] and, as a result, lengthened the time window for the extraneural spread of the disease. A high degree of clinical suspicion and appropriate work-up are necessary to diagnose extraneural metastatic glioblastoma. Molecular and genetic testing may harbor further clues on the etiology of metastatic glioblastoma. Regardless of the etiology, this case illustrates that extraneural metastatic glioblastoma can have an aggressive disease course.

## Conclusions

Glioblastoma, IDH wild type, is an aggressive brain tumor with a poor prognosis despite aggressive treatment regimes. We presented a case of a widely disseminated tumor to multiple vertebrae and bilateral humeri, one of the most extensive metastases documented in the literature. This tumor was found to have an FGFR3-TACC3 fusion, mutations in the TERT gene promoter (124C>T) and the PIK3R1 gene (W583del), as well as loss of CDKN2A/B, FAS, MTAP, and PTEN. Although the genes promoting metastasis of glioblastoma are poorly understood, the mutated genes, in this case, could be explored further for a possible driving cause.
